# The role of social media in public health awareness during times of war in Sudan: snakebites and scorpion stings

**DOI:** 10.1186/s12889-024-19156-8

**Published:** 2024-07-01

**Authors:** Rania M. H. Baleela, Abubakr Mohammad, Sara A. K. Saeed

**Affiliations:** 1 Toxic Organisms Research Centre, Faculty of Science, University of Khartoum, Khartoum, Sudan; 2 Department of Zoology, Faculty of Science, University of Khartoum, Khartoum, Sudan; 3 Conflict and Environment Observatory, West Yorkshire, UK; 4 Natural History Museum, Faculty of Science, University of Khartoum, Khartoum, Sudan

**Keywords:** Social media, Facebook, e-Awareness, eHealth, Citizen science, Data, Envenomation, Snakes, Scorpions, Identification kits, Sudan, Species conservation through positive cultural change

## Abstract

**Background:**

Snakebite envenomation (SBE) and scorpion sting envenomation (SSE) are significant neglected tropical diseases that primarily affect impoverished communities in rural areas of developing nations. A lack of understanding about snake and scorpion species and their distribution exacerbates the disabilities and fatalities caused by SBE and SSE. In Sudan, particularly in regions affected by ongoing conflicts where healthcare resources are scarce, social media platforms offer a cost-effective approach to addressing public health challenges. Our aim in this study is to highlight the benefits of using social media for data collection and health promotion in such environments.

**Methods:**

We present a cost-effective communication and data collection strategy implemented at the Toxic Organisms Research Centre (TORC) of the University of Khartoum, focusing on a Facebook group, “Scorpions and Snakes of Sudan”, as our primary social media platform. Additionally, we discuss the lessons learned and the initial impact of this strategy on enhancing population health literacy.

**Results:**

The group community is composed of ~ 5000 members from 14 countries. During the period from January 2023 to January 2024, we received 417 enquiries about snakes and scorpions belonging to 11 families and composed of 55 species. In addition, 53 other enquiries covered a range of organisms and their tracks (e.g., spiders, skinks, chameleons, foxes, sun spiders, centipedes, lizards, moth larvae, and insect tracks). The first photographic evidence of *Malpolon monspessulanus* in Sudan was via the group activities. The rare species *Telescopus gezirae*, the Blue Nile cat snake, is also documented via the group member’s queries. Recognizing the evolving nature of social media use in public health, we also address the current limitations and evidence gaps that need to be addressed to effectively translate best practices into policy.

**Conclusion:**

In conclusion, utilizing Facebook as an institutional platform to share scientific information in simple Arabic language underscores the proactive roles that citizens, scientists, and public health stakeholders can play in leveraging social media for eHealth, eAwareness, and public health initiatives. This approach highlights the potential for collaborative efforts, particularly during crises, to maximize the benefits of social media in advancing public health.

## Author Summary

Snakebite envenomation (SBE) and scorpion sting envenomation (SSE) are significant public health issues worldwide, particularly affecting vulnerable populations such as children and agricultural workers. SBE alone affects millions of people annually, resulting in thousands of deaths and permanent disabilities. SSE is also a serious concern, with thousands of deaths reported annually but with a dangerous lack of information and in registered incidences, especially in countries such as Sudan. In Sudan, both SBE and SSE are prevalent, with specific regions experiencing higher incidences. To address these challenges, the Toxic Organisms Research Centre (TORC) at the University of Khartoum established a Facebook group, “Scorpions and Snakes of Sudan”, to raise community awareness and collect data on venomous and nonvenomous organisms. The group has attracted nearly 5000 participants, mostly from Sudan, and has received enquiries from nine countries. The data collected through this platform provides valuable insights into the distribution and diversity of snakes and scorpions, aiding in public health interventions and education efforts.

## Background

Snakebite envenomation (SBE) has a significant impact as a disease. Although the exact number of snakebites is uncertain, an estimated 5.4 million people suffer from snakebites annually, resulting in up to 2.7 million cases of envenomation. Each year, approximately 81,000 to 138,000 people die due to SBE, with an additional three times as many individuals sustaining permanent disabilities from these incidents [1]. Venomous snakebites can induce paralysis, potentially leading to respiratory failure, as well as bleeding disorders that may result in fatal haemorrhages. These bites can also cause irreversible kidney failure and tissue damage, leading to permanent disability and necessitating limb amputation. Agricultural workers and children bear the brunt of snakebite impacts, with children often experiencing more severe effects due to their smaller body mass [2]. Given that many snakebite victims are among the most economically active members of society (young, working-age individuals), the disabilities resulting from SBE can impede their ability to earn a living, turning them into lifelong financial burdens for their families. Additionally, snakebite survivors may suffer from disfigurement, posttraumatic stress disorder, stigma, and social exclusion [3].

Another medical problem that requires as much urgent attention as snakebites in Sudan is scorpion sting envenomation (SSE), which is a serious medical emergency that is particularly dangerous for children and elderly people, especially those with respiratory or cardiovascular issues [4]. The global epidemiology of scorpion stings remains poorly understood [5]. It is estimated that between 1.2 and 1.5 million scorpion stings occur annually, resulting in 2,600–3000 deaths [6, 7].

From a clinical standpoint, the primary toxins in Old World (OW) scorpion venoms are α-toxins, which target voltage-gated sodium channels at neurotoxin binding site 3. This action leads to sympathetic excitation and the release of catecholamines, causing transient but life-threatening myocardial damage. Most victims of scorpion stings experience severe local pain, but some, particularly children, may experience systemic envenomation. In the case of most Sudanese buthid species, such as *Androctonus*, *Parabuthus* and *Leiurus*, this envenomation is characterized by cardiovascular and respiratory issues resulting from hypercatecholinaemic myocarditis. Other syndromes associated with OW-SSE include paralysis (*Parabuthus* sp.), coagulopathy (*Nebo hierichonticus* and *Hemiscorpius* species), and local tissue damage, hemolysis, and acute kidney injury (*Hemiscorpius lepturus*) [8].

In Sudan, two retrospective studies analysed hospital-based data on snakebites and scorpion stings [9, 10]. The authors extracted the data for the period from 2014 to 2018 from the annual health statistical reports of the Sudanese Federal Ministry of Health. The snakebite study recorded a total of 63 160 SBEs during 2014–2018, with an average of 12 632 cases/year. The death rate between inpatient patients was 2.5%. The annual incidence is 18–47 cases/100 000 people. Gedarif state recorded the highest incidence (132/100 000 population) of SBE in Sudan, whereas the Northern state had the lowest incidence (5/100 000 population). The 15–24 y age group had the highest risk of snakebite, and males were more likely to be exposed to snakebites than females [9].

Scorpion sting envenomation is a common medical emergency in Sudan. A total of 129,427 victims were envenomed, with a mean of 25,885 cases/year, from 2014 to 2018. Compared with children, more adults were stung. As expected, mortality was greater among children younger than 15 years (*n* = 186, 4.7%) than among older victims (*n* = 56, 1%). SSE was more frequent in males (56%) than in females (44%). The northern state had the highest incidence of SSEs (344 per 100,000 people), followed by the River Nile state (240 per 100,000 people) and Khartoum state (174 per 100,000 people). The mortality rate among hospitalised patients was 2.6% (242/9345), with the northern state accounting for 34% of the total fatalities reported [10].

The World Health Assembly has consistently urged Member States to develop and implement eHealth services, promoting equitable access to digital health benefits, with resolutions emphasising the importance of creating long-term strategic plans [11], resolutions encouraging the use of online and eHealth for eLearning, capacity building and networks [12] and developing infrastructure for health Information and Communication Technologies (ICTs) [13], and ensuring standardisation and interoperability [12]. These strategies have been developed for more than 120 member states, including low- and middle-income countries [14]. In response to these resolutions, the Health Assembly adopted a global strategy on digital health for 2020–2025, aiming to strengthen digital health implementation worldwide. The strategy builds on previous resolutions and global and regional reports, strategies, and recommendations on eHealth and digital health [14]. Social media platforms such as Facebook and Twitter offer promising opportunities to improve population health through eHealth and eAwareness. Social media can encourage citizen participation, use a citizen science approach, optimise health systems, provide an interactive space for science dissemination, support health policies, and promote healthy behaviours. However, these platforms also have limitations that need to be considered, especially in addressing public health SBE and SSE problems.

Facebook is the most favoured social media platform among Sudanese citizens, with more than 74% of social media users using it [15]. Hence, we created the “scorpions and snakes of Sudan” group on January 18^th,^ 2023, with the following objectives: (1) to harness the power of the Facebook platform as a tool for raising community awareness of venomous and nonvenomous animals in Sudan; (2) to use the group as the social media platform of the Toxic Organisms Research Centre; and (3) to use the platform as a means of collecting data concerning the geographic distribution of species for improving the treatment of SBE and SSE in different regions as well as for planning future field work.

## Methods

### Facebook data collection and analyses

The participants uploaded photos of snakes, scorpions, spiders, centipedes, mammals and other organisms. They provided their exact geographical region (country, state and city or village). Group experts, including taxonomists (arachnologist and herpetologists), zoologists, toxicologists, physicians, and biodiversity scientists, provided species names in Latin and in Arabic and local languages if available. Identification cards were also uploaded. All experts’ names and specialities are provided in the Acknowledgement section.

Growth (i.e., total number of members, pending members, approved requests, etc.), engagement (i.e., posts, comments, reactions, active members, top posts, etc.), and participant (i.e., top contributors, age, gender and location) data were then downloaded in MS Excel format and analysed for the period from January 18th, 2023, to January 31^st,^ 2024.

The data of the snakes, scorpions and other animals were collected manually, and the data were manually entered into SM Excel sheets. Data analysis and visualisation were carried out using MS Excel and Zoho Analytics® (https://analytics.zoho.com/). Multiple figures were constructed using https://www.canva.com/.

### Species identification kits

Identification kits were prepared using information from different scientific and clinical resources, including: the Dangerous Snakes of Africa [16], the Reptile Database [17], the Clinical Toxinology Resources [18], Handbook of Amphibians and Reptiles of North-East Africa [19], the scorpion cytogenetic database [20] as well as our personal experiences. Identification kits were regularly uploaded to the group page. The cards were colour coded (red for highly venomous or medically important species, yellow for moderately venomous and green for nonvenomous species). The cards were designed by Rania Baleela using the Canva platform (https://www.canva.com/).

## Results

### Participant demographic and geographical data

**Fig. 1:**
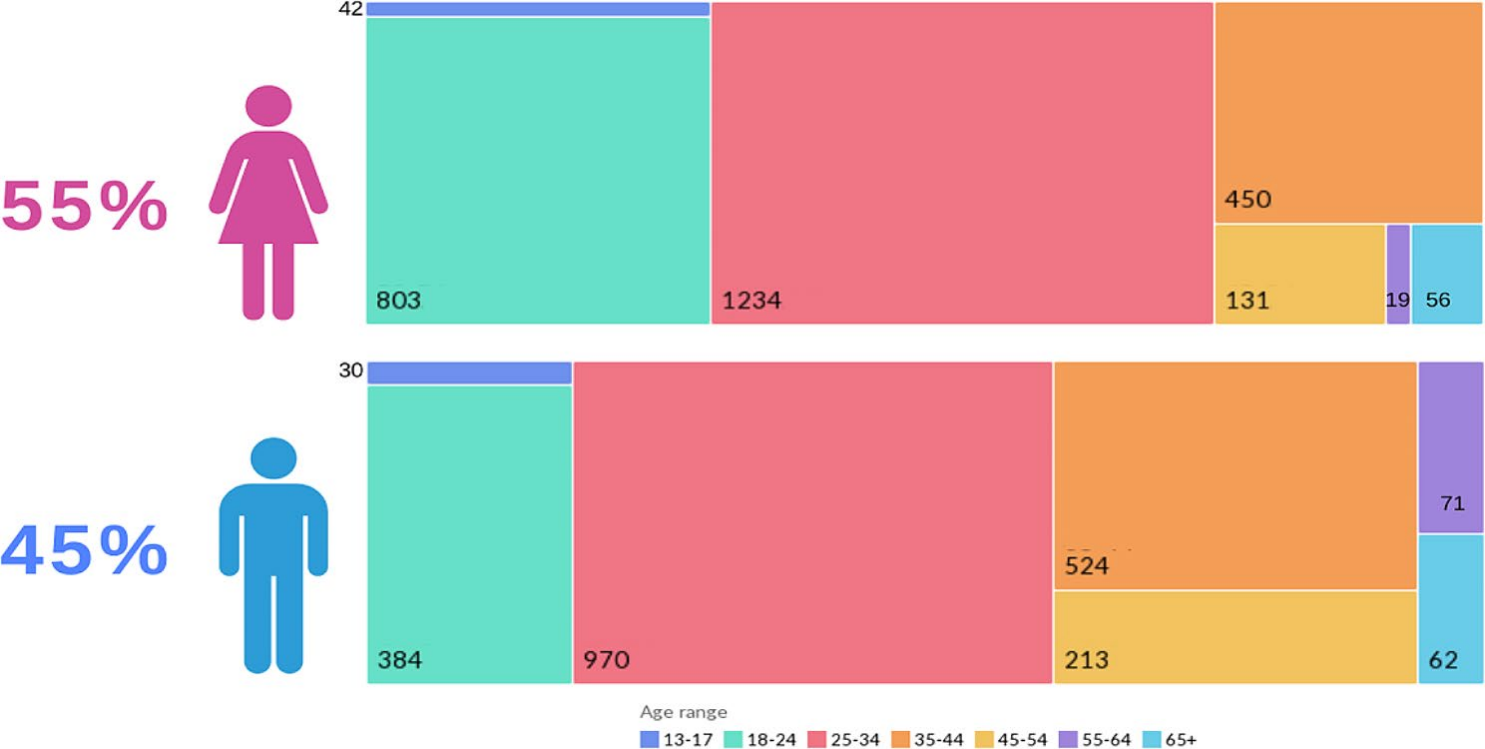
Distribution of participants’ age and sex. **a**) Age group distribution among female participants, **b**) age group distribution among male participants

**Fig. 2:**
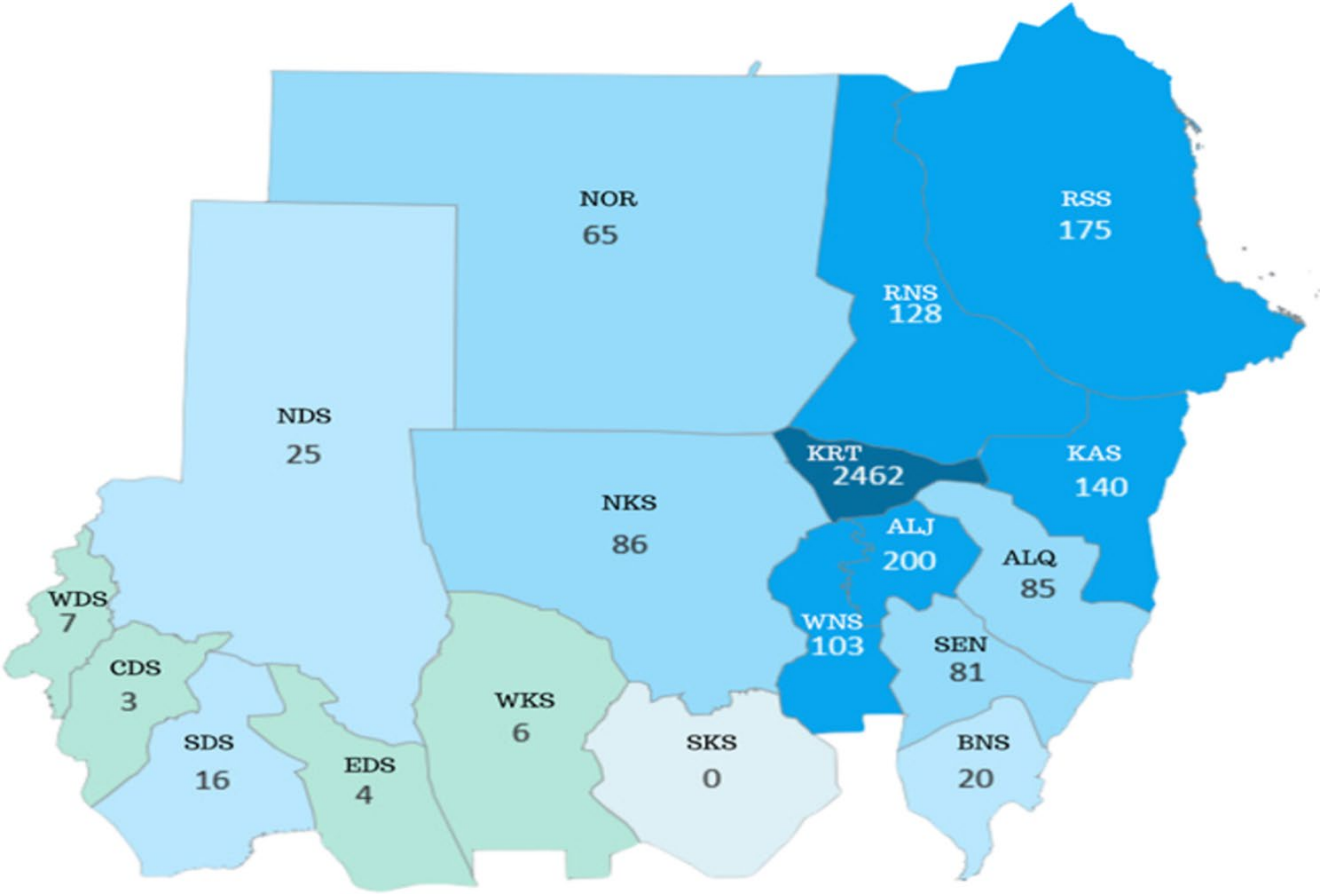
Geographical distribution of participants from each state from the 18 states of Sudan. *Abbreviations* BNS= Blue Nile state, WDS= West Darfur state, NDS= North Darfur state, CDS= Central Darfur state, SDS= South Darfur state, EDS= East Darfur state, WKS= West Kordofan state, SKS= South Kordofan state, NKS= North Kordofan state, ALQ = Gedarif state, ALJ= Gezira state, KAS= Kassala state, KRT= Khartoum state, NOR= Northern state, RSS = Red Sea state, RNS = River Nile state, SEN= Sennar state, WNS= White Nile state

A total of 4989 participants joined the Facebook group between January 2023 and January 2024. There were 2735 females (55%) and 2254 males (45%). An analysis of the age groups revealed that the majority of the participants were in the 25–44 years age group, followed by the 18–24 years and 33–44 years age groups, with a normal distribution (Fig. 1). Geographically, we have participants on every continent except for South America and Antarctica. The majority of participants came from Sudan, especially the capital Khartoum (Figure 2).

### Enquiries about snakes and scorpions

Between January 2023 and January 2024, we received a total of 417 enquiries from 55 snake and scorpion species belonging to 11 families from 9 countries. During this time, 56 identification cards were produced in Arabic.

Of the 417 enquiries, 208 (49.88% of all received enquiries) involved scorpions. Two scorpion families were present, accounting for 30.9% of the observed species diversity. The two families were (1) Buthidae, represented here by 15 species and contributing to 97% of the surveyed scorpions, and (2) Scorpionidae, represented here by 2 species and contributing to 2.88% of the surveyed scorpions.

**Fig. 3:**
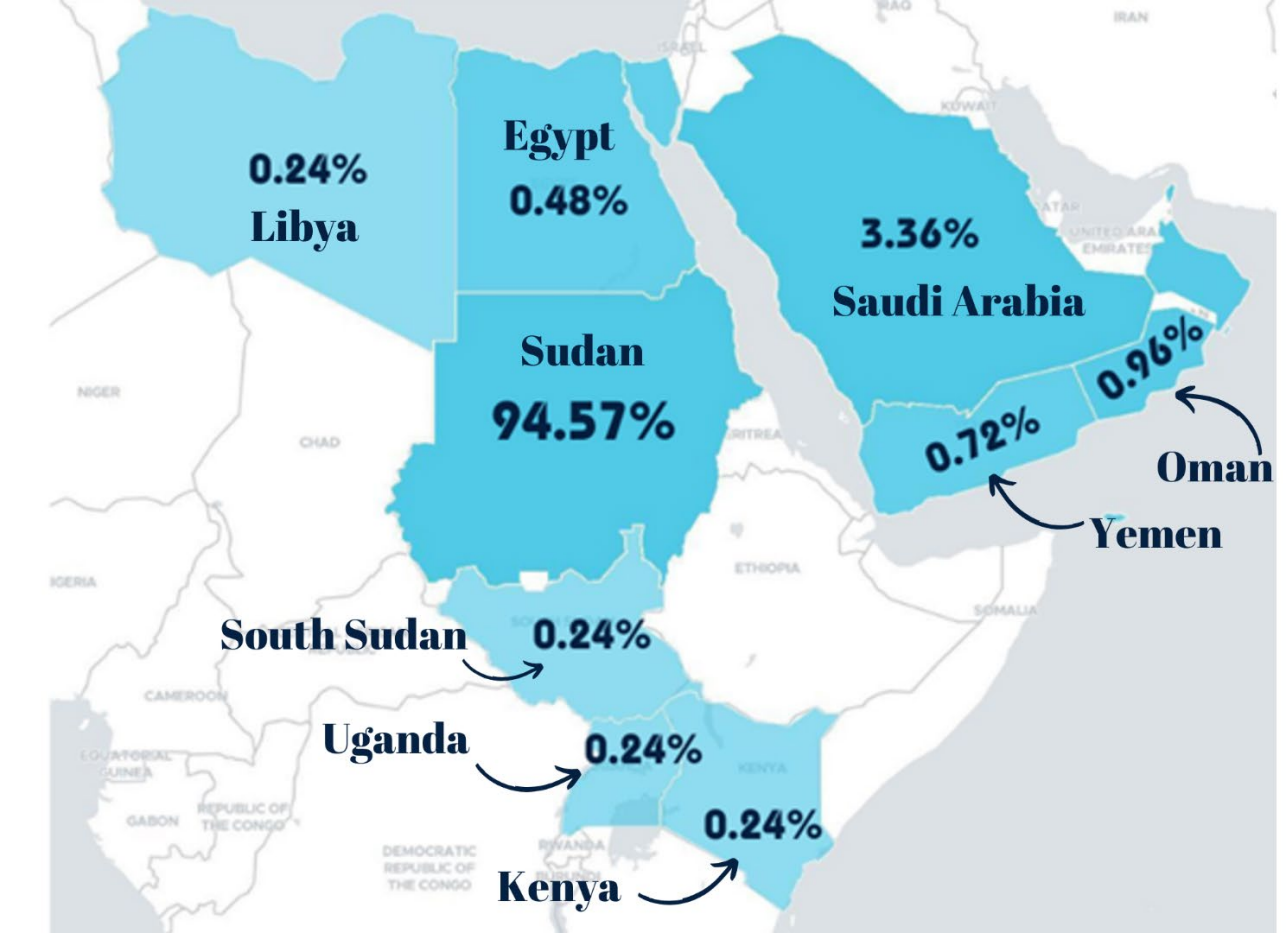
Geographical distribution of received enquiries from 9 countries: ordered from the country with the highest to the lowest enquiries: Sudan, Saudi Arabia, Oman, Yemen, Egypt, Libya, Uganda, South Sudan, and Kenya. A single inquiry was received from India as well

There were 209 enquiries about snakes (50.11% of all received enquiries). Nine families related to snakes were identified, accounting for 38 unique species and 69.1% species diversity, and the families were (1) Viperidae (5 species), (2) Elapidae (6 species), (3) Atractaspididae (2 species), (4) Psammophiidae (8 species), (5) Colubridae (10 species), (6) Pythonidae (1 species), (7) Boidae (2 species), (8) Leptotyphlopidae (2 species) and (9) Lamprophiidae (2 species) (Figure 3; Table 1).

**Table 1:** Species, including those of medical importance, reported in the enquiries received per state in Sudan and per country in other countries

No.	Species	Sudanese States																		Other countries								Medical importance
		Abyei Area	Blue Nile	East Darfur	North Darfur	South Darfur	Gedarif	Gezira	Kassala	Khartoum	West Kordofan	North Kordofan	South Kordofan	Northern	Red Sea	River Nile	Sennar	Unknown	White Nile	Egypt	Kenya	Libya	Oman	Saudi Arabia	South Sudan	Uganda	Yemen	
A. Snakes																												
**1. Family: Viperidae**																												
1	***Bitis arietans***																		X									H
2	***Echis pyramidum***	X					X	X	X	X	X			X			X											H
3	*Echis burkini*																							X				H
4	*Echis carinatus*																							X				H
5	***Cerastes cerastes***									X				X														H
**2. Family: Elapidae**																												
6	***Naja nubiae***							X	X	X							X											H
7	***Naja nigricollis***												X				X											H
8	***Naja haje***							X									X			X								H
9	*Naja subfulva*																									X		H
10	*Naja arabica*																										X	H
11	*Dendroaspis polylepis*																				X							H
**3. Family: Atractaspididae**																												
12	***Atractaspis watsoni***			X				X																				H
13	***Atractaspis phillipsi***		X				X	X								X	X		X									H
	**4. Family: Psammophiidae**																											
14	***Malpolon monspessulanus***¶										X																	MO
15	***Malopolon moilensis***													X		X												MO
16	***Psammophis sudanensis***							X	X	X				X		X	X		X									MO
17	***Psammophis mossambicus***			X			X	X									X	X										MO
18	***Psammophis sibilans***																X											MO
19	***Psammophis rukwae***							X	X																			MO
20	*Psammophis schokari*																							X				MO
21	***Psammophis spp.***							X																X				MO
**5. Family: Colubridae**																												
22	***Platyceps florulentus***						X	X	X	X				X					X									N
23	*** Platyceps rhodorhachis***																						X					N
24	***Philothamnus semivariegatus***														X													M
25	***Dispholidus typus***					X																			X			H
26	***Dasypeltis sahelensis***			X																								N
27	***Spalerosophis diadema***							X				X			X	X				X				X				N
28	***Telescopus obtusus***							X																				M
29	***Telescopus geziraeǂ***																X											M
30	*Macroprotodon cucllatus*																					X						M
31	*Unknown*																										X	N/A
	**6. Family: Leptotyphlopidae**																											
32	***Myriophlis sp.***							X		X						X		X	X									N
**7. Family: Lamprophiidae**																												
33	***Boaedon fuliginosus***															X	X											N
34	***Boaedon longilineatus***																		X									N
**8. Family: Boidae**																												
35	***Eryx colubrinus***							X			X			X	X	X												N
36	***Eryx muelleri***			X				X																				N
**9. Family: Pythonidae**																												
37	***Python sebae***							X	X	X				X		X	X	X										N, P
**Total number of snakes**		**1**	**1**	**4**	**0**	**1**	**5**	**57**	**15**	**17**	**3**	**1**	**26**	**15**	**5**	**14**	**26**	**17**	**8**	**2**	**1**	**1**	**1**	**8**	**1**	**1**	**3**	
**Medically important count (%)**		1	1	2 (50)	0	1	3 (60)	10 (18)	5 (33)	5 (29)	1 (33)	0	7 (27)	4 (27)	0	4 (29)	7 (27)	0	3 (38)	1 (50)	1	0	0	6 (75)	1	1	2 (67)	
**Number of species reported**		**1**	**1**	**4**	**0**	**1**	**4**	**16**	**6**	**7**	**3**	**1**	**1**	**7**	**3**	**8**	**11**	**3**	**6**	**2**	**1**	**1**	**1**	**5**	**1**	**1**	**2**	
**No.**	**B. Scorpions**																											
**1. Family: Buthidae**																												
1	***Androctonus amoreuxi***									X		X		X	X	X		X										H
2	*Androctonus crassicauda*																							X				H
3	*Apistobuthus sp.*																							X				H
4	*Buthacus spp.*																							X				M
5	*Compsobuthus spp.*																							X				M
6	***Compsobuthus werneri***							X	X	X				X		X	X	X	X									M
7	*Hottentotta jayakri*																						X					MO
8	***Hottentotta minax***							X		X						X*	X											MO
9	***Hottentotta niloticus***							X		X			X				X											MO
10	***Hottentotta spp.***								X								X											MO
11	***Hottentotta trailini***						X	X																				MO
12	***Leiurus quinquestriatus***							X						X		X	X	X	X									H
13	***Neobuthus sudanensis***																		X									M
14	***Parabuthus abyssinicus***							X	X					X		X		X										H
15	*Parabuthus liosoma*																							X				H
**2. Family: Scorpionidae**																												
16	*Heterometrus sp.*																						X					M
17	***Pandinurus sudanicus***				X	X	X											X										M
**Total number of scorpions**		**0**	**0**	**0**	**1**	**1**	**2**	**67**	**9**	**15**	**0**	**1**	**9**	**26**	**1**	**52**	**9**	**8**	**6**	**0**	**0**	**0**	**6**	**4**	**0**	**0**	**0**	
**Medically important**		0	0	0	0	0	1 (50)	5 (7)	2 (22)	3 (20)	0	1	0	3 (12)	1	3 (6)	0	0	1 (17)	0	0	0	2 (33)	4 (100)	0	0	0	
**Number of species reported**		**0**	**0**	**0**	**1**	**1**	**2**	**6**	**3**	**4**	**0**	**1**	**1**	**4**	**1**	**5**	**5**	**5**	**3**	**0**	**0**	**0**	**2**	**5**	**0**	**0**	**0**	

Bolditalicsare the species reported from Sudan. Abbreviations: X = reported in inquiries, H = highly venomous and potentially life threatening, M = mildly venomous, MO = moderately venomous, N = nonvenomous, P = potentially life threatening, N/A = not applicable, ¶= first record from Sudan, *= first record from this state, ǂ= rare species

Furthermore, 53 additional enquiries covered various organisms, including spiders, skinks, chameleons, foxes, sun spiders, centipedes, lizards, moth larvae, and insect tracks. Some members also contributed to awareness efforts by uploading video content about venomous snakes, while others shared their experiences in protecting their homes from snake invasions using local materials.

As expected, the majority of these enquiries originated from Sudan, totalling 390 enquiries (93.53% of all enquiries related to snakes and scorpions). Sudan accounted for 28 of the 37 snake species (76.68%) and 8 of the 17 scorpion species (47.06%). For further information, please refer to Table 1, which summarizes enquiries from other countries.

### Patterns of enquiries received from Sudan

**Fig. 4:**
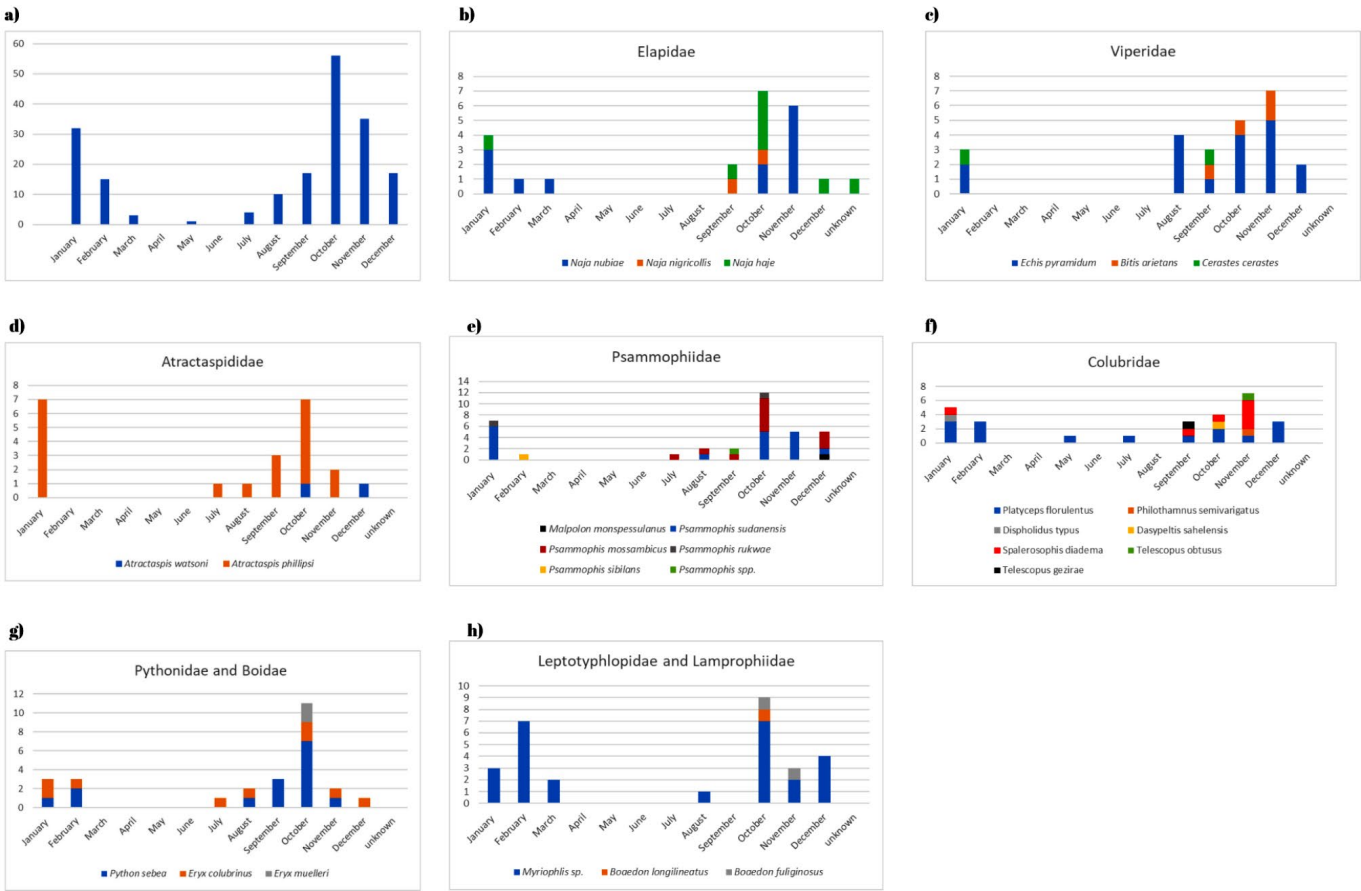
Chronology of snake enquiries from Sudan: **a**) for all species, **b**) for elapids, **c**) for vipers, **d**) for burrowing asps, **e**) for sand racers, **f**) for colubrids, **g**) for python and boas and h) for blind and house snakes

**Fig. 5:**
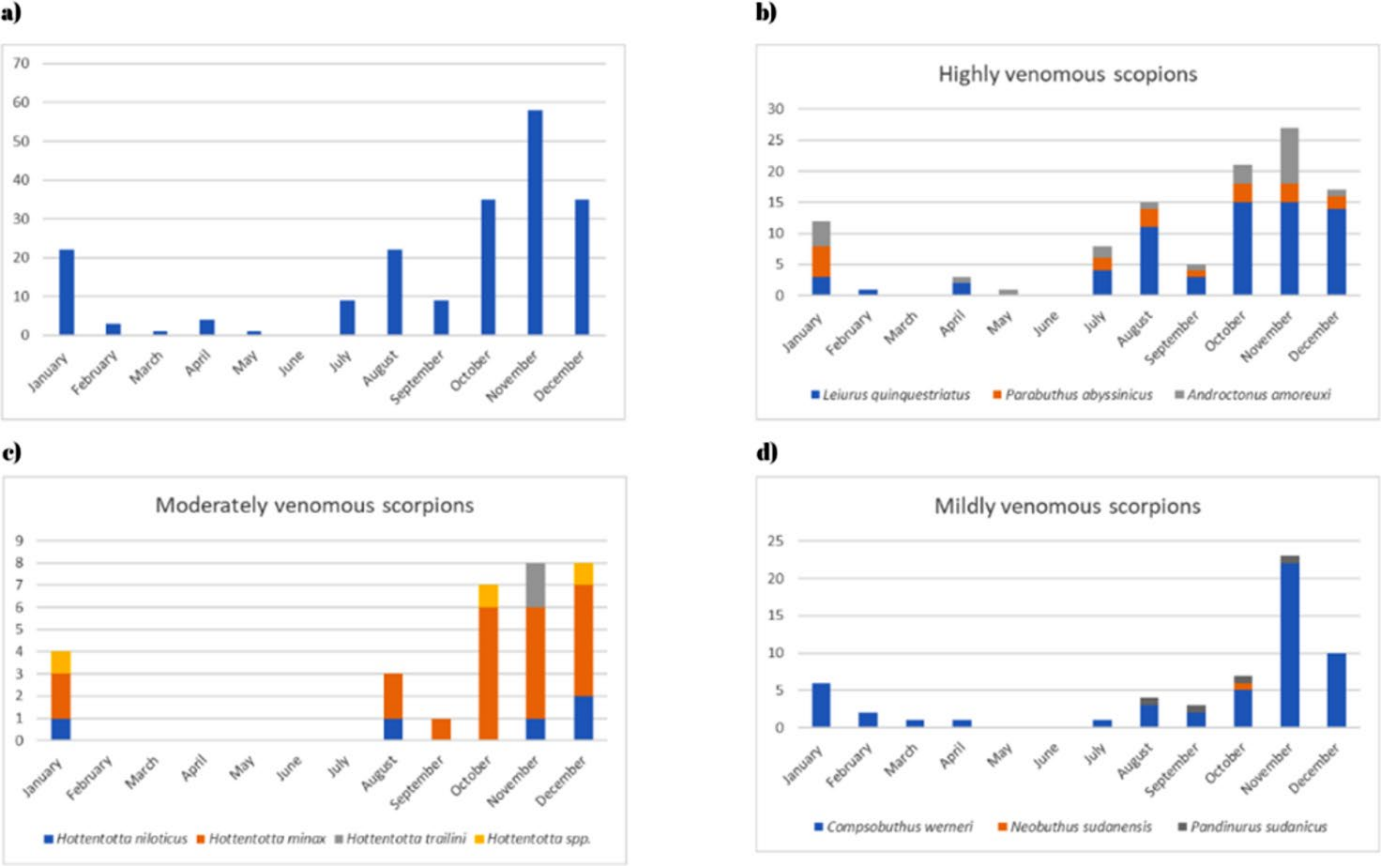
Chronology of scorpion’s enquiries from Sudan: **a**) all species, **b**) highly venomous scorpions, **c**) moderately venomous scorpions and **d**) mildly venomous scorpions

**Fig. 6:**
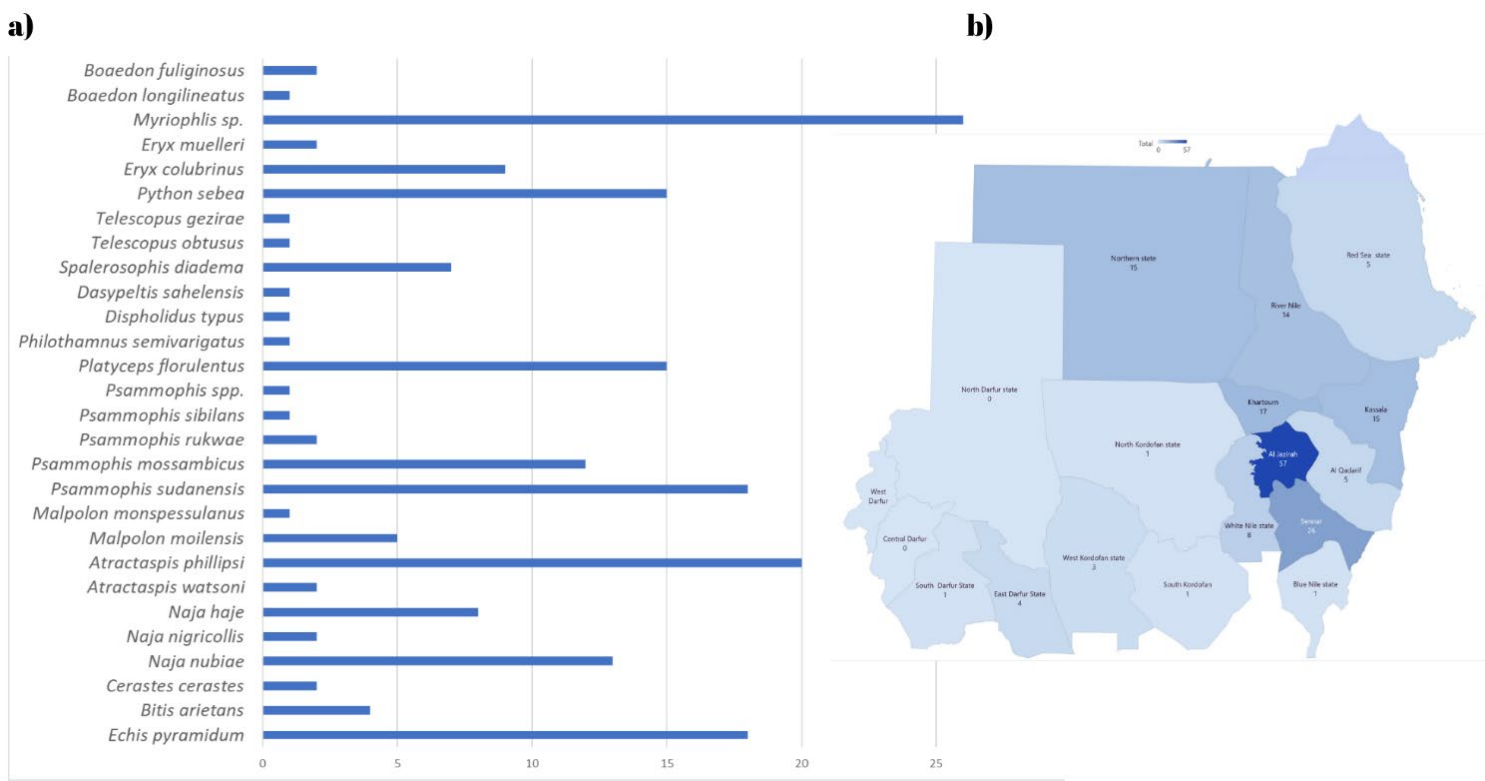
Diversity of snakes in Sudan: **a**) 28 species of snakes enquired about with *Myriophils spp*. showing the highest number of enquiries followed by *Atractaspisphillipsi, Echis pyramidum and Psammophis sudanensis*, **b**) The number of enquiries about snakes received from each of the 18 Sudanese states

**Fig. 7:**
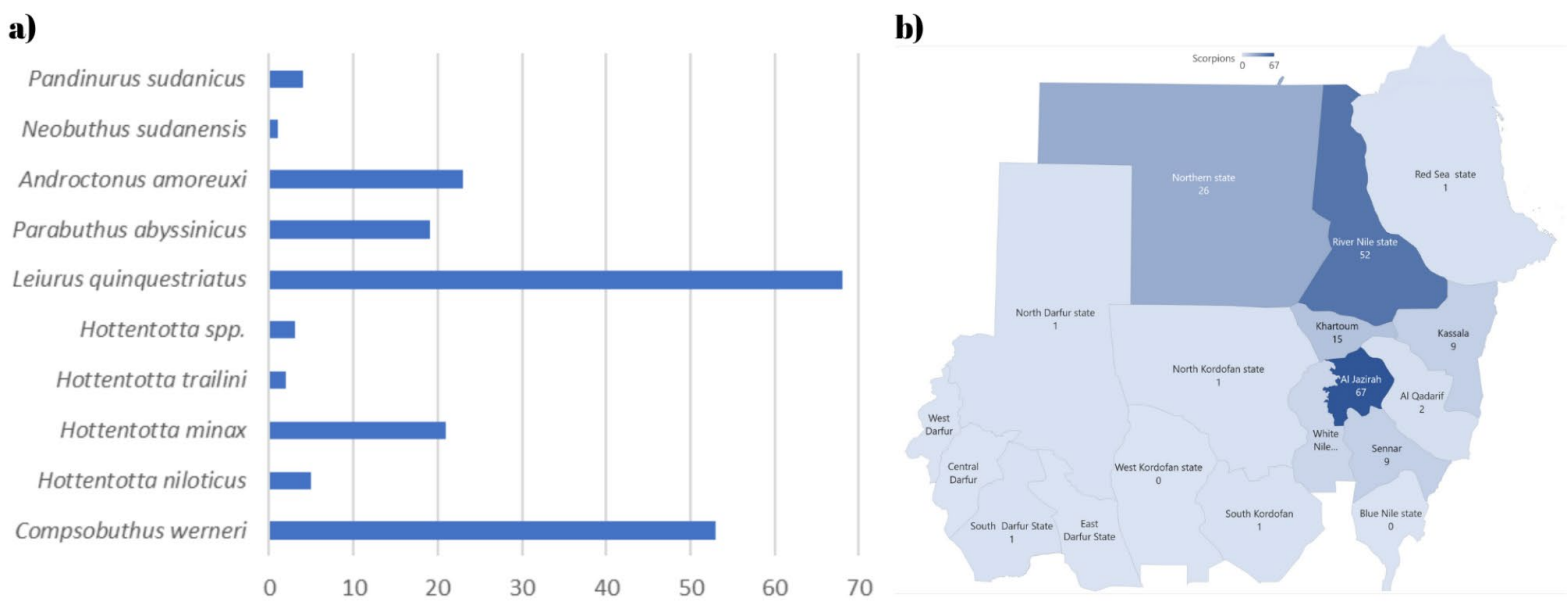
Scorpion Diversity in Sudan: **a**) We received enquiries about 10 scorpion species, with the highest number of enquiries being about the highly venomous death stalker (*Leiurus quinquestriatus*), followed by the mildly venomous species *Compsobuthus werneri*, **b**) The number of enquiries about scorpions received from each of the 18 Sudanese states

According to the chronological pattern, the period from July through March had the greatest number of enquiries about snakes, with the peak occurring in October (Fig. 4). However, viperids and colubrids peak in November, followed by October. For scorpions, the peak in enquiries was observed in November, followed by October and December. However, there were two smaller peaks in August and January (Fig. 5). A total of 20 medically important snake species from Sudan were identified. In Gezira state, 17 different snake species were reported from 32 unique locations, 10 of which are medically important. Sennar had enquiries about 13 species, 7 of which were medically important. The River Nile had enquiries about 8 species, 4 of which are medically important, while Khartoum State and the Northern State had enquiries about 7 species each, with 5 and 4 being medically important, respectively. The White Nile River had enquiries about 6 species, 3 of which were medically important, and East Darfur had enquiries about 4 species, 2 of which were medically important. There were 3 species each in Gedarif, West Kordofan, and the Red Sea, all of which were medically important in Gedarif, none in the Red Sea state, and 1 in West Kordofan state. Additionally, there was 1 inquiry each from the Blue Nile, South Kordofan, South Darfur, and Abyei administrative areas, all of which are medically important, and one non medically important inquiry from North Kordofan (Table 1). The diversity of the organisms reported here per state enquiries is provided in Figs. 6 and 7. In Gezira state, enquiries were made about 6 different scorpion species, while the River Nile and Sennar states had enquiries about 5 species each. Khartoum state and the Northern state each had 4 species, the White Nile had 3 species, and Gedarif had 2 species. Additionally, North Darfur, North Kordofan, the Red Sea, South Darfur, and South Kordofan each had enquiries about 1 species. There were also 5 species for which enquiries were received from unknown states (Table 1). The scorpion species with the greatest number of enquiries was the highly venomous *Leiurus quinquestriatus*, followed by the mildly venomous species *Compsobuthus werneri*.

**Fig. 8:**
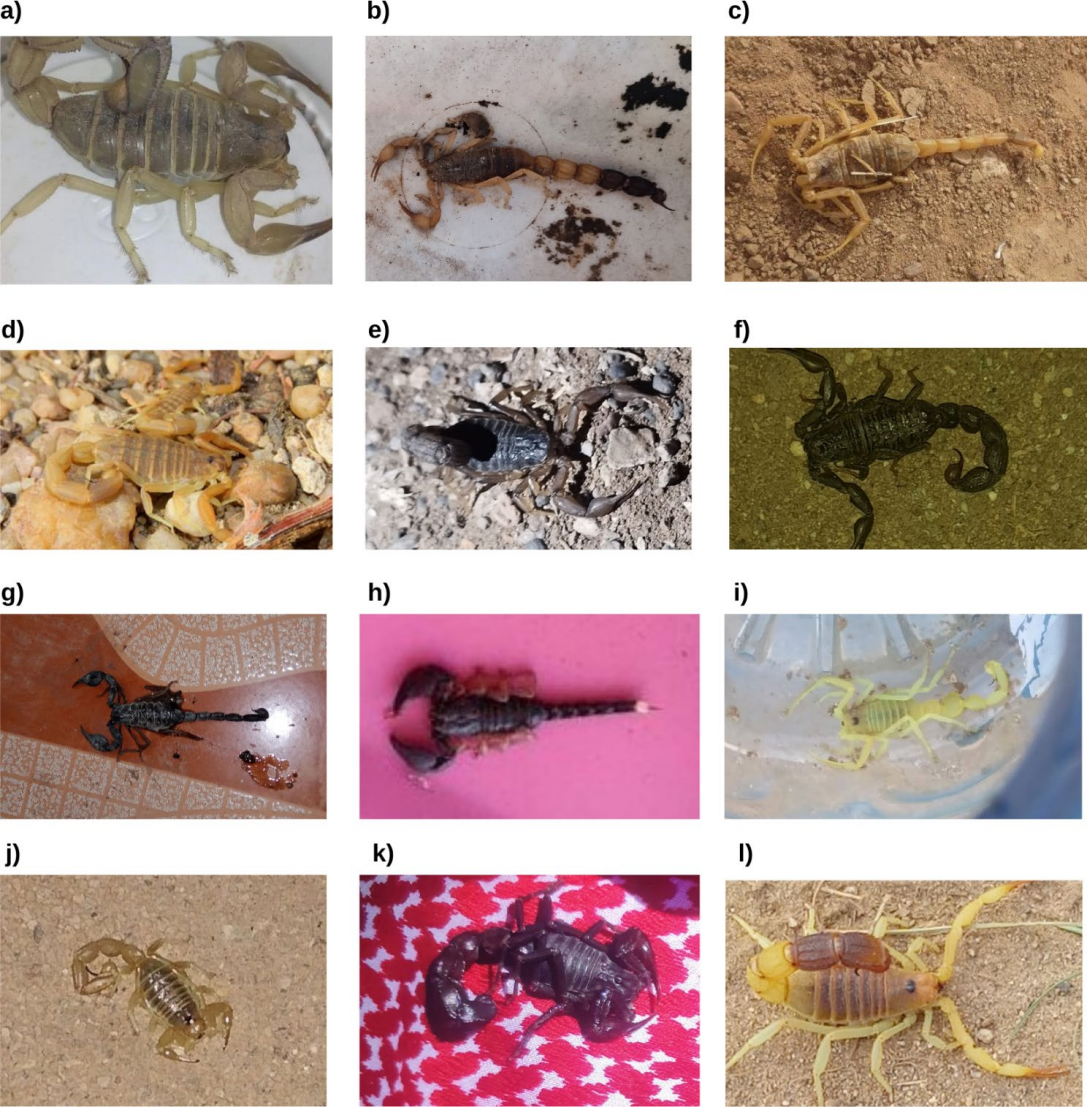
Example of scorpion species photographs submitted to the Facebook group for identification: **a**) *Androctonus amoreuxi* from Sudan, **b**) *Parabuthus abbysinicus* from Sudan, **c**) *Leiurus quinquestriatus* from Sudan, **d**) *Compsobuthus werneri* from Sudan, **e**) *Hottentotta niloticus* from Sudan, **f**) *Hottentota trailini* from Sudan, **g**) *Heterometrus sp.* from Oman, **h**) *Opisthacanthus sp.* from India, **i**) *Apistobuthus pterygocercus* from Oman, **j**) *Buthacus sp.* from Saudi Arabia, **k**) *Androctonus crassicauda* from Saudi Arabia and **l**) *Parabuthus liosoma* from Saudi Arabia

**Fig. 9:**
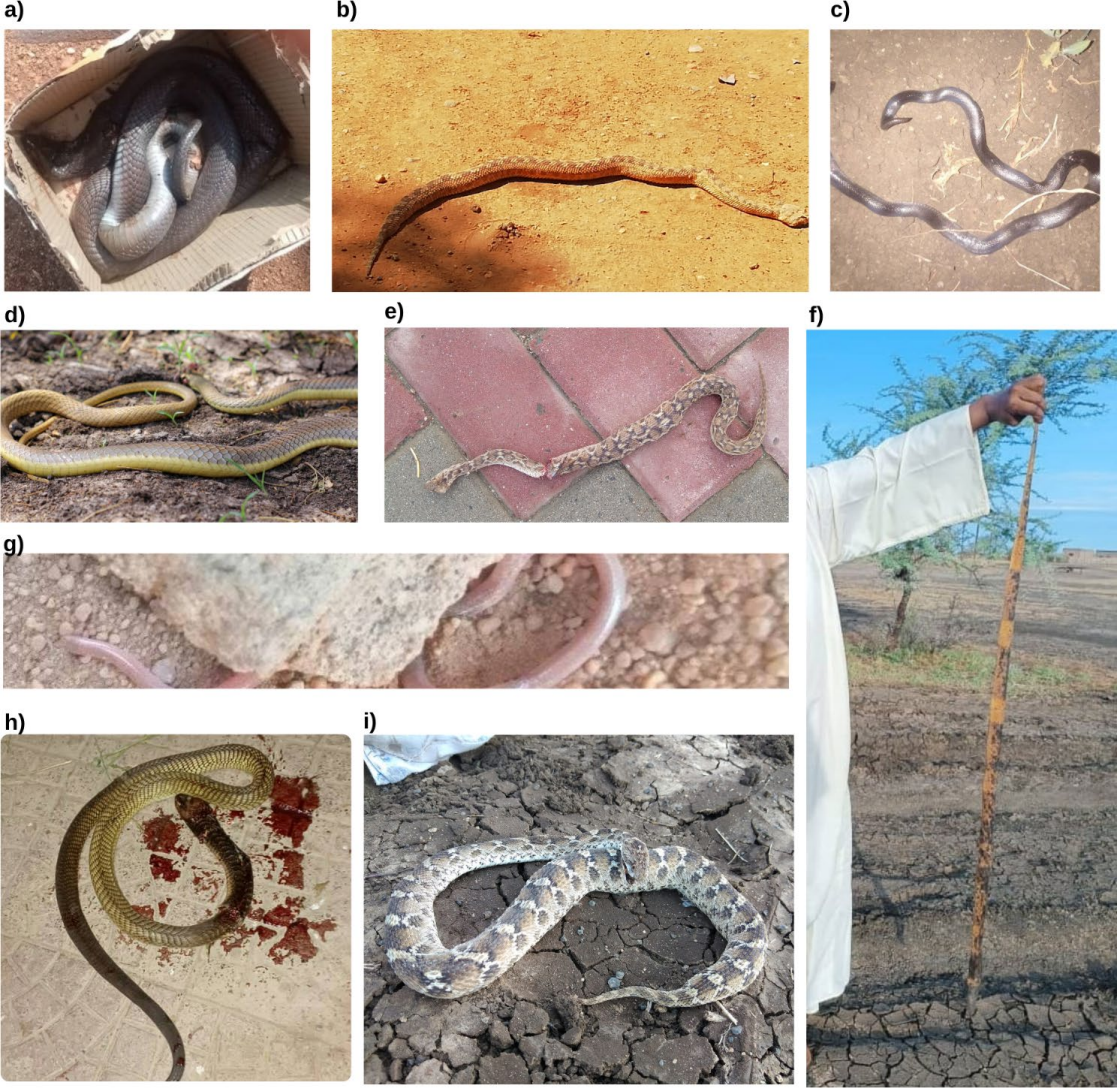
Example of the snake species submitted to the Facebook group for identification: **a**) *Naja nigricollis* from Sudan, **b**) *Cerastes cerastes* from Sudan, **c**) *Atractaspis phillipsi* from Sudna, **d**) *Psammophis mossambicus *from Sudan, e) *Echis carinatus* from Saudi Arabia, **e** **f**) *Naja haje* from Sudan, **g**) *Myriophils sp* from Sudna, **h**) *Naja arabica* from Yemen, and **i**) *Echis pyramidum* from Sudan

**Fig. 10:**
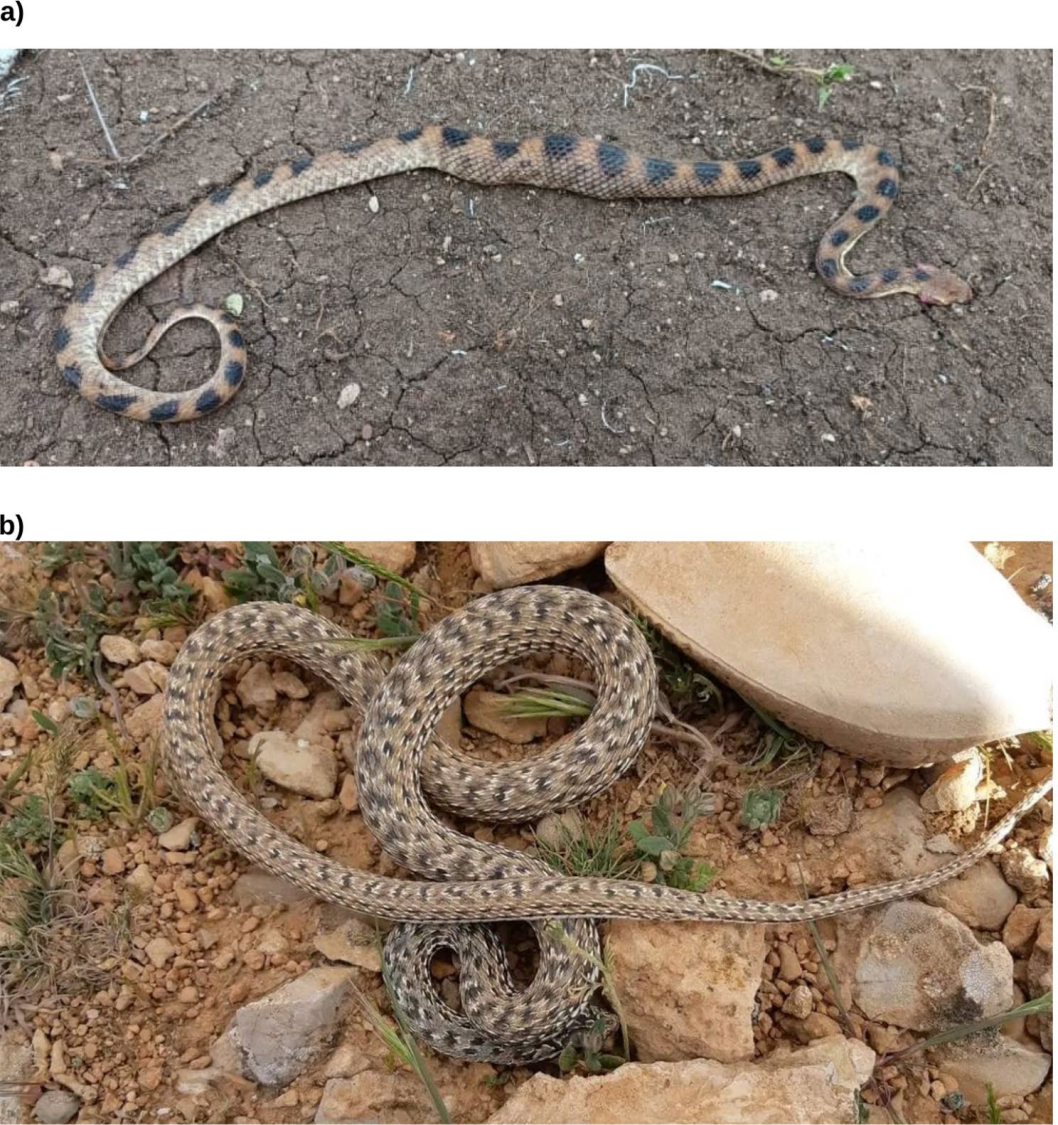
Rare species and first records from Sudan: **a**) observation of the rare Blue Nile cat snake (*Telescopus gezirae*) and **b**) first record of Montpellier Snake (*Malpolon monspessulanus*) juvenile from Sudan

**Fig. 11:**
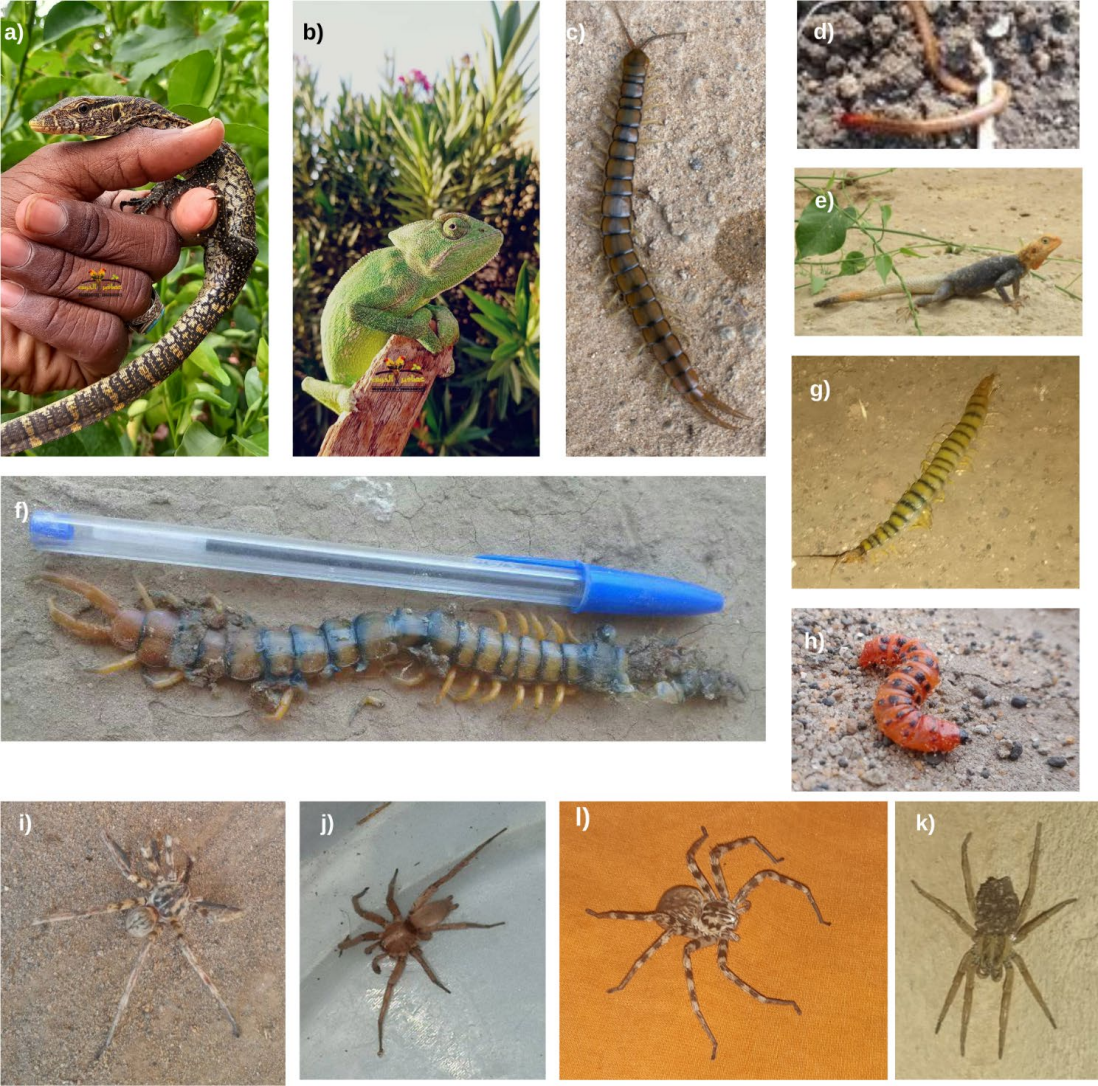
Example of other organisms submitted to the group for identification: **a**) juvenile Nile monitor lizard (*Varanus niloticus*), **b**) Basilisk chameleon (*Chamaeleo africanus*), **c**) and **f**) tiger centipede (*Scolopendra sp.*) **d**) soil centipede, **e**) male Malaba Rock Agama/Finch’s Agama (*Agama finchi*), **g**) *Scolopendra* sp., **h**) *Cactoblastis cactorum *larva, **i-k***Eusparassus *sp. **l**) Wolf spider (*Family: Lycosidae*)

Samples of the photos submitted for identification can be found in Figs. 8, 9, 10 and 11. A rare species, the Blue Nile Cat snake *Telescopus gezirae*, was reported from Sennar state, while a juvenile of the species *Malpolon monspessulanus* was recorded for the first time in Sudan from West Kordofan state (Fig. 10), with the observation documented in iNaturalist under the *Sudan Fauna and Flora Project*. Additionally, there are records of endemic species, such as *Parabuthus abbysinicus* from the Red Sea state, reported for the first time in states where they have never been reported before.

### Identification key development for raising community eAwareness

**Fig. 12:**
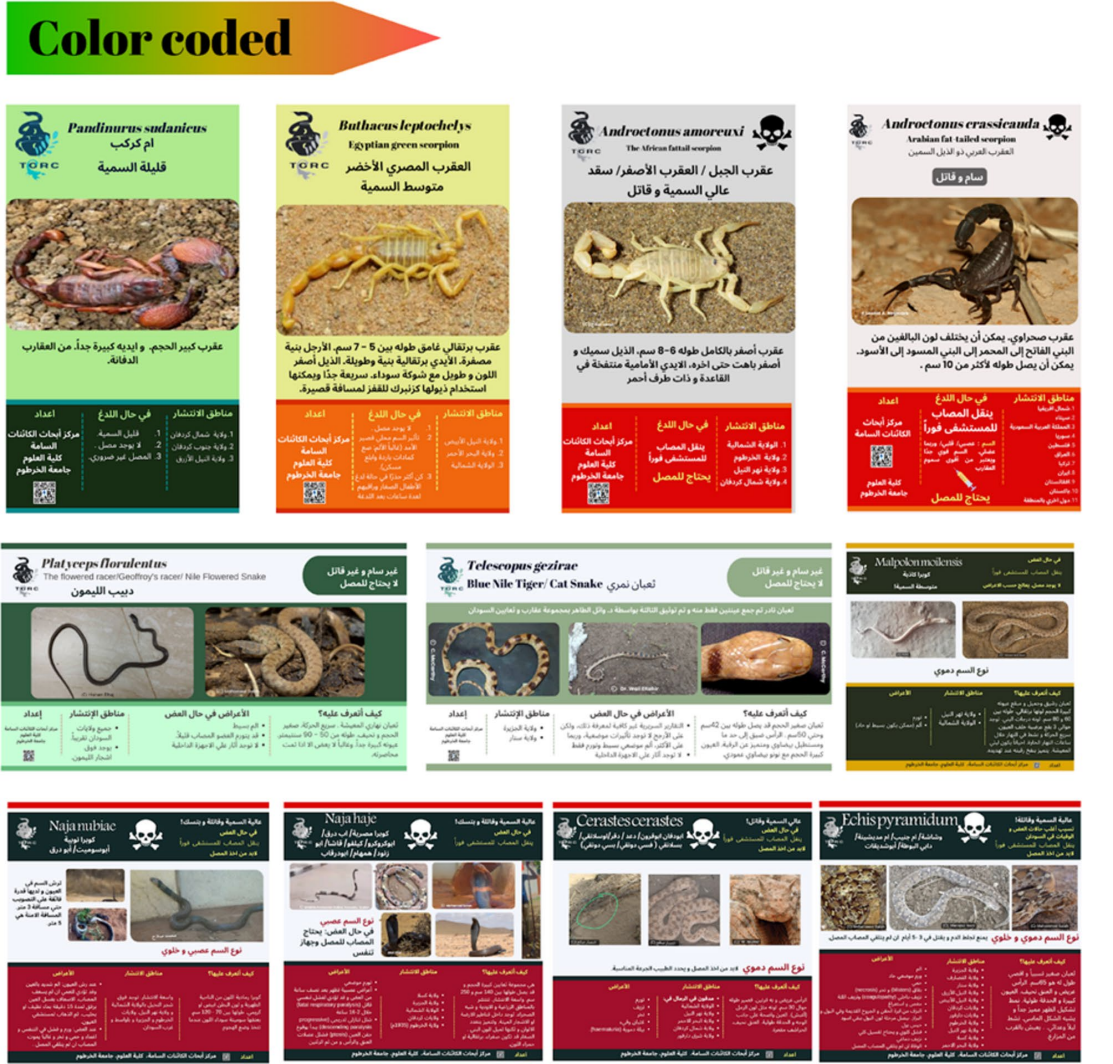
Examples of produced ID cards are color-coded from red to green, with red indicating highly venomous or medically important species, yellow for moderately venomous and green for nonvenomous species

**Fig. 13:**
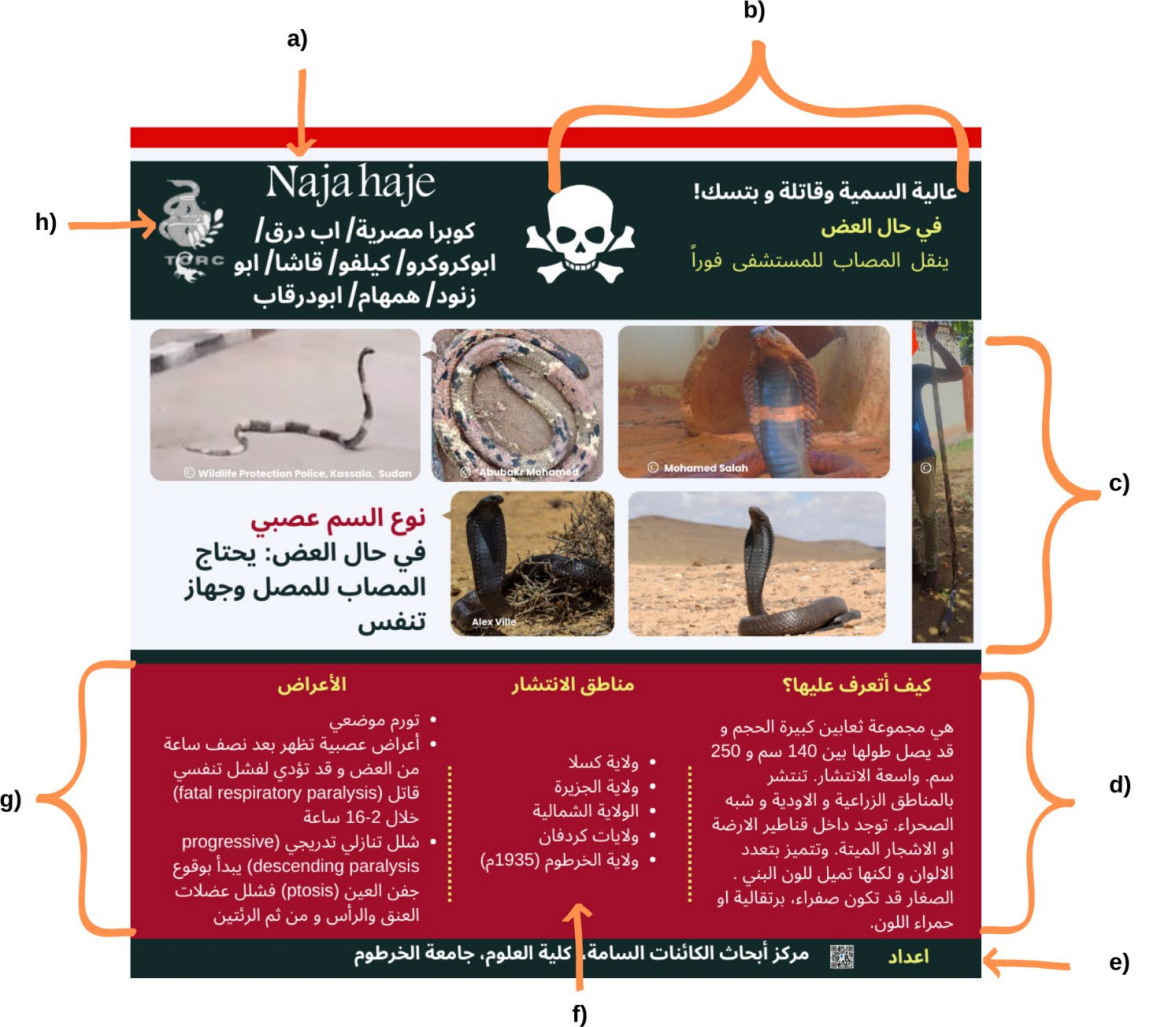
The detailed structure of an identification card, clockwise: each card is composed of the following sections: **a**) Scientific and local names, **b**) level of dangerousness, venom type and immediate first aid required, **c**) Photos representing the species, **d**) description, **e**) developed by TORC, **f**) geographical distribution, **g**) symptoms, and **h**) The centres’ logo

In the past year (2023), we developed and shared 56 identification cards with the online community. These cards, the first of their kind in Arabic, aim to help individuals identify venomous and nonvenomous organisms. Each card features at least one photo of the organism, geographic distribution information, a layman’s description without scientific terminology, and symptoms in case of envenomation, whether from a snakebite (SBE) or scorpion sting (SSE). The cards are colour-coded, with red indicating highly venomous or medically important species, yellow indicating moderately venomous and green indicating nonvenomous organisms. An example of the produced ID kits is shown in Fig. 12, and the structure of each individual ID card is detailed in Fig. 13.

### Conserving species through positive cultural change

When our group first started, the majority of the photos we received were of dead animals. There was a prevailing belief that all snakes or lizards were venomous and should be killed. This was especially true for species such as the sandfish skink (*Scincus scincus*) and sand boas (*Eryx colubrinus* and *Eryx muelleri*), where early posts consistently showed dead animals.

However, this has changed recently. Through our eAwareness efforts, we were able to educate people about these species being non venomous and harmless. As a result, we have seen a shift in behaviour, with people now submitting photos and videos of living specimens. They are also seeking advice on safe ways to return these animals to their natural habitats, and we have been able to provide them with instructions to do so.

### Lessons learned and the initial impact

Based on our experience through the last year (2023), the lessons learned include the following: (1) Community engagement is crucial: engaging with local communities is essential for understanding their perspectives, needs, and fears regarding venomous organisms. This engagement can help dispel myths, raise awareness about snake safety, and promote conservation efforts; (2) Research and education go hand in hand: conducting scientific research on venomous organisms not only advances scientific knowledge but also provides valuable information for education and awareness programs. These findings can be used to develop effective strategies for mitigating the impact of SSEs and SBE and conserving biodiversity; (3) Knowledge is crucial to improve medical outcomes: gathered data can guide medical personnel by helping them identify the medically important venomous species indigenous to their geographic region, leading to improved medical treatment plans and public health outcomes; (4) Collaboration is key: collaboration with local and international partners, including experts, physicians on the ground and abroad and academic institutions, can enhance research and conservation efforts. These collaborations can provide access to resources and expertise. (5) Communication is essential: communicating scientific findings in a clear and accessible manner is important for increasing awareness and promoting behavioural change. Effective communication can help bridge the gap between scientific research and local communities (e.g., ID cards and conversations with people online). (6) Adaptability is necessary: dealing with venomous organisms requires adaptability and flexibility, as field conditions and challenges can vary widely. Researchers and conservationists must be prepared to adjust their strategies based on the local context (e.g., the war in Sudan complicated the already complex situation and adapting ourselves to have other means to collect data while raising awareness is priceless); (7) persistence pays off despite facing challenges such as limited resources and logistical difficulties, persistence and dedication can lead to meaningful results; and (8) ultimately, establishing a Facebook page could have simplified the data download process, potentially eliminating the need for manual data entry. Moreover, this manuscript, based on TORC experience, demonstrates the importance of perseverance in tackling complex issues such as SSEs, SBE and biodiversity conservation during a crisis.

## Discussion and conclusions

Several publications have shown that there is a strong association between the peak of snakebite envenomation and humanitarian crises such as conflict, displacement, floods, and the migration of impoverished agricultural workers [21–25]. The high burden of snakebites in the Central African Republic, Ethiopia, South Sudan, and Yemen seems to coincide with the disastrous humanitarian situation in three of these countries (CAR, South Sudan, and Yemen) and with impoverished worker migration in Ethiopia. Similar conditions are found in northern Nigeria and several other areas of the world affected by humanitarian crises and migration [21]. As of February 2024, nearly 8 million Sudanese people have been displaced as a direct effect of the current war. This includes more than 6 million people displaced within Sudan and more than 1.5 million people who fled to neighbouring countries. This conflict exacerbated many of Sudan’s existing challenges, including ongoing conflicts, disease outbreaks, economic and political instability and climate emergencies [26].

As anticipated, the majority of participants and enquiries originated from Sudan. In addition, the bulk of those from Sudan came from the capital Khartoum. However, it is important to note that this may not accurately represent the submitter’s geographic location, as Facebook shares initial data that members used to create an account. In reality, many people from Khartoum had to evacuate the capital and were displaced elsewhere due to the eruption of the war on April 15th, 2023.

Between January 2023 and January 2024, we received a total of 417 enquiries from 9 countries. This highlights a significant gap in strictly scientific content provided for the public in the Arabic language, which we endeavoured to address. Additionally, we observed that the number of enquiries about scorpions was almost equal to that about snakes. This indicates the need for focused research on scorpion stings and their epidemiology, possibly elevating them to a neglected tropical disease (NTD).

Our chronological analysis may be useful for predicting and forecasting the appropriate times to stock antivenoms in specific geographic regions, especially in countries facing crises and resource scarcity, such as Sudan. Contrary to the common belief that scorpion stings peak in March during the summer, our data indicate that scorpions are more prevalent during the winter, aligning with observations shared by some members. However, no hospital data are currently available to support or refute this observation. Regarding snakes, enquiries started in July through March and peaked in October. Notably, vipers and colubrids exposures peaked in November, followed by October.

We uploaded the collected data into iNaturalist under the Sudan Fauna and Flora Project for broader dissemination and community sharing. To our knowledge, this is the first publication to record the presence of the colubrid species *Malpolon monspessulanus* in West Kordofan state, Sudan. Additionally, we documented the rare observation of the Blue Nile Cat snake *Telescopus gezirae* (Broadley 1994), which is known from only 2 specimens [27].

Our ID cards, which we developed, are currently the only available resources for scientific information regarding venomous organisms in the Arabic language directed towards the public.

Among the ~ 2000 scorpion species described, the venom of 50 species is dangerous for humans, and most of these species belong to the family Buthidae. The most medically important species belong to the following genera: *Hottentotta, Buthus, Tityus, Leiurus, Androctonus, Parabuthus, Centruroides* and *Mesobuthus* [28].

We report here enquiries about the highly venomous species *Leiurus quinquestriatus, Androctonus amoreuxi, Androctonus crassicauda, Apistobuthus* sp., *Parabuthus liosoma* and *Parabuthus abyssinicus.* In addition to enquiries about the moderately medically important species *Hottentotta jayakri, Hottentotta minax, Hottentotta niloticus, Hottentotta trailini* and another *Hottentotta* spp. members were not identified at the species level. Moreover, we investigated the mildly venomous scorpions *Neobuthus sudanensis, Buthacus* spp., *Compsobuthus werneri, Compsobuthus* spp., *Heterometrus* sp. and *Pandinurus sudanicus*.

Social media is a powerful platform for introducing scientific evidence to the general public and thus serves as a powerful, cost-effective tool for people of all ages to learn and share knowledge daily (i.e., for e-education and e-awareness). They can also be used for public health surveillance, optimising policy interventions, identifying vulnerable groups geographically, and designing policies that consider individuals’ interactions within communities.

Conserving species through positive cultural change involves engaging local communities, raising public awareness, and conducting scientific research. These efforts are crucial for addressing human‒wildlife conflict, garnering support for protection measures, and informing conservation strategies.

Involving local communities in conservation is vital because they often possess valuable traditional knowledge about the environment and wildlife. This knowledge can complement scientific, biomedical and medical research and help develop culturally sensitive and sustainable conservation strategies. Engaging with communities allows conservationists to build trust, gain insights into local perspectives and needs, and promote conservation awareness. This approach has been successful in various projects, such as community-based conservation efforts for snow leopards in the Himalayas [29].

Educating the public about biodiversity and conservation is essential for fostering a culture of conservation. Public awareness campaigns can help individuals understand the value of wildlife and ecosystems as well as the impacts of human activities on them. By raising awareness, people can be encouraged to support conservation initiatives, make sustainable lifestyle choices, and advocate for conservation policies [30, 31], thereby contributing to enhanced public health.

Initially, our group predominantly received photos of deceased animals. There was a widespread belief that all snakes or lizards were venomous and should be eliminated. This perception was particularly strong for species such as the sandfish skink (*Scincus scincus*) and sand boas (*Eryx colubrinus* and *Eryx muelleri*), as early posts consistently depicted deceased animals.

However, there has been a recent shift in this mindset. Through our eAwareness efforts, we have been able to educate people about these species as nonvenomous and harmless. Consequently, we have observed a change in behaviour, with individuals now submitting photos and videos of living specimens. They are also seeking advice on safe methods to return these animals to their natural habitats, and we have been able to provide them with guidance for doing so.

Based on our experience last year (2023), several key lessons have emerged regarding the management of snakebite envenomation (SBE), scorpion sting envenomation (SSE), and biodiversity conservation. These lessons emphasise the importance of community engagement, research, collaboration, communication, adaptability, persistence, and the potential benefits of utilising social media platforms such as Facebook

Engaging with local communities is crucial for understanding their perspectives, needs, and fears regarding venomous organisms. This engagement can help dispel myths, raise awareness about snake safety, and promote conservation efforts [32].

In addition, collecting and analysing data on venomous organisms not only advances scientific knowledge but also provides valuable information for education and raising awareness, ultimately benefiting public health.

Our findings can be used to develop effective strategies for mitigating the impact of SBE and SSE and conserving biodiversity [33]. Currently, the primary challenge in educating people about snakebites is to determine the antivenom requirements at an operational, local level. To achieve this goal, it is crucial to collect epidemiological data, which will enable the seasonal anticipation of snakebite numbers, species involved, and geographic locations [34]. While this objective has been largely achieved in the Americas [33, 35], the availability of such data is limited in Asia, with some existing data in countries such as India [36] but not enough to provide a comprehensive view of the situation [37].

Analyses of African data have highlighted that the problem of snakebites in Africa is greatly underestimated [38], a finding supported by initial epidemiological analyses from national health registries [39]. Addressing these data gaps and improving the collection and analysis of epidemiological data are essential for developing effective strategies to prevent and treat snakebites. Moreover, collaborating with local and international partners, including experts, physicians, and academic institutions, can enhance research and conservation efforts. These collaborations can provide access to scientific and medical resources expertise. This was clearly evident during our last year (i.e. year 2023) of experience.

Communicating scientific findings in a clear and accessible manner is important for raising awareness and promoting behavioural change. Effective communication can help bridge the gap between scientific research and local communities [40], and our aim was to address this gap by distributing very clear scientific messages in local slang or Arabic languages for the community to understand.

Managing venomous organisms requires adaptability and flexibility, as field conditions and challenges can vary widely. Researchers and conservationists must be prepared to adjust their strategies based on the local context. Thus, during the war in Sudan and because conducting surveys became almost impossible, we used this platform as a pilot data collection platform that will help us direct our future surveys when the situation calms on the ground.

Establishing a Facebook page could simplify the data download process, potentially eliminating the need for manual data entry.

In conclusion, these lessons underscore the importance of a holistic approach. This approach integrates community engagement, research, collaboration, communication, adaptability, persistence, and the utilization of social media platforms. These are key in addressing the challenges posed by SBE, SSE and biodiversity conservation. Despite challenges such as limited resources and logistical difficulties, persistence and dedication can lead to meaningful results. Collectively, these different factors can improve public health.

The Facebook group “Snakes and Scorpions of Sudan” serves as a valuable platform for raising awareness and the potential for collecting data from remote geographical locations. However, this study also has limitations. One limitation is the potential for misinformation to spread. Since the group relies on user-generated content, there is a risk that inaccurate information about snakes and scorpions could be shared, leading to misunderstandings and potentially dangerous situations.

**Fig. 14:**
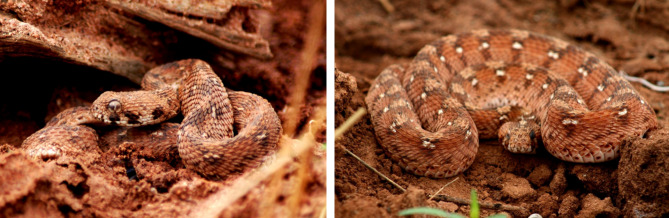
*Echis romani* from west of Kadugli in South Kordofan state, Sudan

**Fig. 15:**
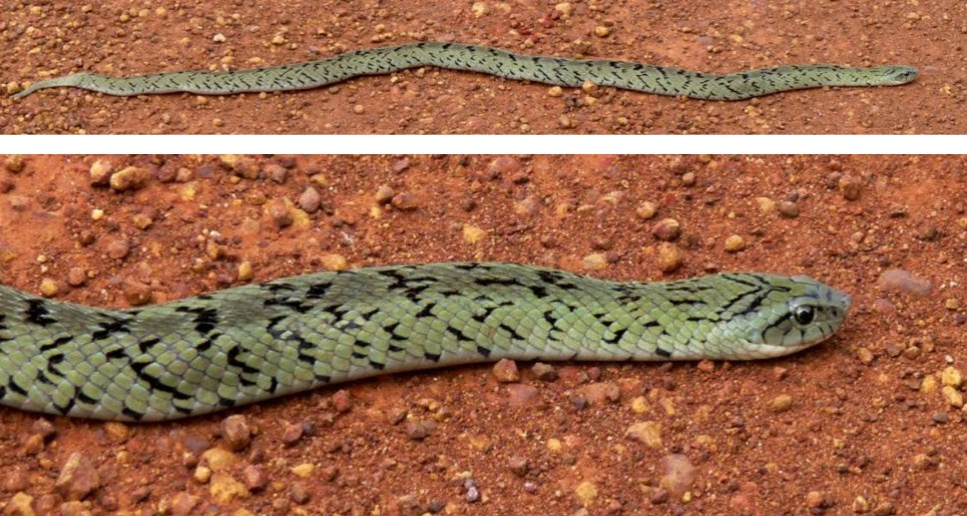
*Causus resimus* from Sudan

Another limitation is the accessibility of the group. Not all Sudanese people have access to Facebook or the internet, so the information shared in the group may not reach everyone who could benefit from it. This could particularly affect individuals living in rural or remote areas where Internet access is limited. For example, we know that other venomous species such as *Echis romani* (Fig. 14) and *Causus resimus* (Fig. 15) [19] are present in regions where internet connection may not be available and hence were not reported here.

Additionally, the group may face challenges in reaching and engaging with certain demographics, such as older adults or those with lower levels of education, who may be less likely to use Facebook or participate in online discussions.

Overall, while the Facebook group “Snakes and Scorpions of Sudan” is a valuable resource, it is important to recognize its limitations and consider complementary approaches to reach a broader audience and ensure the accurate dissemination of information.

In this paper, we show the usefulness of social media as a means of citizen science that is useful for raising awareness and for collecting data from remote geographical locations. Our findings align somewhat with the statistics reported in the previous decade Federal Ministry of Health annual reports, which we have also analysed (unpublished data).

## Data Availability

No datasets were generated or analysed during the current study.

## References

[1] WHO. Snakebite envenoming, World Health Organization, 12 September 2023. [Online]. Available: https://www.who.int/news-room/fact-sheets/detail/snakebite-envenoming. [Accessed 21 February 2024].

[2] Waiddyanatha S, Silva A, Siribaddana S, Isbister G (2019). Long-term effects of Snake Envenoming. Toxins (Basel).

[3] Harrison R, Hargreaves A, Wagstaff S, Faragher B, Lalloo D (2009). Snake Envenoming: a Disease of Poverty. PLoS Negl Trop Dis.

[4] El-Aziz F, El Shehaby D, Elghazally S, Hetta H (2019). Toxicological and epidemiological studies of scorpion sting cases and morphological characterization of scorpions (Leiurusquin Questriatus and Androctonus crassicauda) in Luxor, Egypt. Toxicol Rep.

[5] Santos M, Silva C, Neto B, Grangeiro Júnior C, Lopes V, Teixeira Júnior A, Bezerra D, Luna J, Cordeiro J, Júnior J, Lima M (2016). Clinical and epidemiological aspects of scorpionism in the world: a systematic review. Wilderness Environ Med.

[6] Chippaux J, Goyffon M (2008). Epidemiology of scorpionism: a global appraisal. Acta Trop.

[7] Ebrahimi V, Hamdami E, Moemenbellah-Fard M, Jahromi S (2017). Predictive determinants of scorpion stings in a tropical zone of south Iran: use of mixed seasonal autoregressive moving average model. J Venom Anim Toxins Incl Trop Dis no.

[8] Amr Z, Abu Baker M, Al-Saraireh M, Warrell D (2021). Scorpions and scorpion sting envenoming (scorpionism) in the Arab countries of the Middle East. Toxicon.

[9] Khalid H, Azrag RS (2021). Retrospective hospital-based study on snakebite envenomation in Sudan. Trans R Soc Trop Med Hyg.

[10] Khalid H, Siyam ME, a MEMOE, Azrag RS (2023). Scorpion stings envenomation in Sudan: a retrospective study of hospital-based incidence. Toxicol Commun.

[11] WHO, Assembly. Fifty-eighth World Halth, resolutions and decisions annex. Resolution WHA58.28. eHealth, World Health Organization, 16–25 May 2005. [Online]. Available: https://apps.who.int/gb/ebwha/pdf_files/WHA58-REC1/english/A58_2005_REC1-en.pdf. [Accessed 22 February 2024].

[12] WHO, Resolution. WHA66.24. eHealth standardization and interoperability. In: Sixty-sixth World Health, World Health Organization, 20 – 27 May 2013. [Online]. Available: https://apps.who.int/gb/ebwha/pdf_files/WHA66/A66_26-en.pdf. [Accessed 22 February 2024].

[13] WHO, Resolution. WHA71.7 Digital Health, Seventy-First World Health Assembly, 26 May 2018. [Online]. Available: https://iris.who.int/bitstream/handle/10665/279505/A71_R7-en.pdf?sequence=1. [Accessed 22 February 2024].

[14] WHO. Global strategy on digital health 2020–2025, World Health Organization, 2021. [Online]. Available: https://www.who.int/docs/default-source/documents/gs4dhdaa2a9f352b0445bafbc79ca799dce4d.pdf. [Accessed 22 February 2024].

[15] Statcounter SMS, Sudan. Statcounter, 2023. [Online]. Available: https://gs.statcounter.com/social-media-stats/all/sudan. [Accessed January 2023].

[16] Spawls S, Branch B (2020). The dangerous snakes of Africa.

[17] Uetz P, Freed P, Aguilar R, Reyes F, Kudera J, Hošek J. The Reptile Database, 2023. [Online]. Available: https://reptile-database.reptarium.cz/. [Accessed January through December 2023].

[18] Department T, Resources CT. Toxinology Department, Women’s & Children’s Hospital, University of Adelaide, 2001–2018. [Online]. Available: http://www.toxinology.com/index.cfm. [Accessed 7 01 2024].

[19] Spawls S, Mazuch T, Mohammad A (2023). Handbook of amphibians and reptiles of North- East Africa.

[20] Schneider M, Mattos V, Cella D. The scorpion cytogenetic database, 2023. [Online]. Available: www.arthropodacytogenetics.bio.br/scorpiondatabase.

[21] Alcoba GP, Vatrinet R, Singh S, Nanclares C, Kruse A, Boer M, Molfino L, Ritmeijeri K (2022). Snakebite envenoming in humanitarian crises and migration: a scoping review and the Médecins sans Frontières experience. Toxicon X.

[22] Amr Z, Abu Baker M, Warrell D (2020). Terrestrial venomous snakes and snakebites in the arab countries of the Middle East. Toxicon.

[23] Tameru K (2006). Snake bite envenomation at Aysha refugee camp health center. Ethiop Med J.

[24] Rashid A, Adnan M (2009). Pakistan’s refugees face uncertain future. Lancet.

[25] Steegemans I, Sisay K, Nshimiyimana E, Gebrewold G, Piening T (2022). Treatment outcomes among snakebite patients in north-west Ethiopia—A retrospective analysis. PLoS Negl Trop Dis.

[26] UNHCR. Sudan Crisis Explained, The United Nations Refugee Agency, 16 February 2024. [Online]. Available: https://www.unrefugees.org/news/sudan-crisis-explained/. [Accessed 23 March 2024].

[27] Crochet P, Jens B, Thomas W, Philippe G, Jean-Francois T (2008). Systematic status and correct nomen of the western north African cat snake: Telescopus tripolitanus (Werner, 1909) (Serpentes: Colubridae), with comments on the other taxa in the dhara-obtusus group. Zootaxa no.

[28] Borges A, Graham M, Calvete J (2016). Phylogenetics of scorpions of Medical Importance. Venom Genomics and Proteomics. Toxinology.

[29] Jackson R, Mishra C, McCarthy T, Ale S (2010). Snow leopards: conflict and conservation. Biology and Conservation of Wild Felids.

[30] Xu W, Chong J, Li J (2020). An exploration of the influence of Panda Awareness Week campaign on pro-environmental behavior. J Clean Prod.

[31] Wittemyer G, Northrup JM, Blanc J, Douglas-Hamilton I, Omondi P, Burnham KP. Illegal killing for ivory drives global decline in African elephants, *Proceedings of the National Academy of Sciences*, vol. 111, no. 36, pp. 13117–13121, 2014. 10.1073/pnas.1403984111 PMC4246956 25136107

[32] Sodeinde OA, Afolabi BM (2018). Snakebite as a cause of deaths in children: still an issue in Africa. Ann Trop Paediatr.

[33] Chippaux JP (2017). Snakebite envenomation turns again into a neglected tropical disease!. J Venom Anim Toxins Including Trop Dis.

[34] Chippaux J (2008). Estimating the global burden of snakebite can help to improve management. PLoS Med.

[35] Chippaux J (2017). Incidence and mortality due to snakebite in the Americas. PLoS Negl Trop Dis.

[36] Mohapatra B, Warrell D, Suraweera W, P B, Dhingra N. and e. al., Snakebite mortality in India: a nationally representative mortality survey, *PLoS Negl Trop Dis*, vol. 5, no. 4, p. e1018., 2011. 10.1371/journal.pntd.0001018 PMC3075236 21532748

[37] Gutierrez J, Williams D, Fan H, Warrell D. Snakebite envenoming from a global perspective: Towards an integrated approach, *Toxicon*, vol. 56, no. 7, pp. 1223–1235, 2010. 10.1016/j.toxicon.2009.11.020 19951718

[38] Chippaux J (2011). Estimate of the burden of snakebites in sub-saharan Africa: a meta-analytic approach. Toxicon.

[39] Habib AG, Kuznik A, Hamza M, Abdullahi MI, Chedi BA, Chippaux J, Warrell D (2015). Snakebite is under appreciated: Appraisal of Burden from West Africa. PLoS Negl Trop Dis.

[40] de Lange E, Sharkey W, Castelló y Tickell S, Migné J, Underhill R, Milner-Gulland E. Communicating the biodiversity crisis: from warnings to positive engagement. Trop Conserv Sci 2022;15:2022. 10.1177/19400829221134893.

